# Factors associated with fetal karyotype in spontaneous abortion: a case-case study

**DOI:** 10.1186/s12884-022-04491-8

**Published:** 2022-04-14

**Authors:** Qinghua Xu, Ying Chan, Yun Feng, Baosheng Zhu, Bicheng Yang, Shu Zhu, Lingyun Su, Li Zou, Na Feng, Yan Li

**Affiliations:** 1 School of Public Health, Kunming Medical University, No. 1168, Chunrongxi Road, Yuhua Street, Chenggong, Kunming, 650500 Yunnan China; 2 Kunming Children’s Hospital (Children’s Hospital Affiliated to Kunming Medical University), No. 288, Qianxing Road, Kunming, 650228 Yunnan China; 3 The First People’s Hospital of Yunnan Province, No. 157, Jinbi Road, Kunming, 650034 Yunnan China

**Keywords:** Chromosomal abnormality, Spontaneous abortion, Homocysteine, Genetic factor, Environment

## Abstract

**Background:**

Most embryos that spontaneously abort during early pregnancy are found to have chromosomal abnormalities. The purpose of this study is to explore the factors involved in chromosome aberrations during embryogenesis.

**Methods:**

A case-case study was performed to compare the risk factors for spontaneous abortion with and without embryo chromosome aberration. A total of 160 cases of spontaneous abortion were enrolled from a tertiary general hospital in Kunming. KaryoLite BACs-on-Beads (KL-BoBs) and fluorescence in situ hybridization (FISH) were employed to determine chromosomal constitution of abortion chorion villus samples. Maternal serum levels of homocysteine (Hcy) were detected by high performance liquid chromatography-tandem mass spectrometry (HPLC-MS/MS). Information about clinical background and environmental exposure was collected through a self-designed questionnaire. To identify the inherited chromosomal abnormalities, couples with chromosomal abnormalities in abortus were recalled for karyotyping.

**Results:**

The overall rate of chromosomal abnormalities was 62.5% (100/160, KL-BoBs combined with FISH) including 51.9% (83/160) aneuploidies, 6.3% (10/160) polyploidies, and 4.4% (7/160) structural abnormalities. Only one case of structural abnormality was found to be inherited from maternal balanced translocation. Compared to abortus with normal karyotype, abortus with abnormal karyotype showed a positive association with parental age and elevated maternal serum homocysteine (Hcy) level, but negative association with previous miscarriage and perceived noise.

**Conclusions:**

Embryonic chromosomal aberrations accounted for the majority of spontaneous abortion cases. A combination of internal and external factors may induce spontaneous abortion through fetal chromosomal aberrations or other pathogenic mechanisms.

**Supplementary Information:**

The online version contains supplementary material available at 10.1186/s12884-022-04491-8.

## Introduction

Spontaneous abortion is a common complication in human reproduction. An estimated 60% of spontaneous abortions occur before or after implantation (termed preclinical losses), while 10–15% are confirmed by ultrasound or histological evidence (termed clinical miscarriage) [1, 2]. Most clinical miscarriages occur during the first trimester, and the leading cause of miscarriage is embryonal chromosomal abnormalities [3, 4]. Although parental chromosomal structure abnormalities are the main genetic factor leading to recurrent miscarriages, the prevalence of chromosomal abnormalities in the affected couples is relatively low (2.78–4.1%) [5–7]. Few studies have investigated the parental karyotype of sporadic miscarriage [3]. The frequency of balanced rearrangements in the general population is very low (0.4%) [1, 2, 8].

Apart from inheritable factors, a variety of maternal factors have been found to be related to spontaneous abortion or embryonic chromosomal aberrations, including age, reproductive history, and immune or endocrine dysfunction [1–3, 9]. In addition, elevated maternal serum level of homocysteine was shown to increase the risk of fetal loss and stillbirth [10]. Supplementation of folic acid can decrease the concentration of homocysteine and reduce the risk of pregnancy loss [11, 12]. Polymorphisms in folate metabolizing genes are associated with chromosome breaks and fetal chromosomal aneuploidy [13, 14].

Apart from maternal factors, embryonic chromosomal abnormalities may be attributable to abnormal gametogenesis in father. Sperm DNA fragmentation index tends to increase with paternal age [15, 16]. Patients with high levels of sperm DNA damage showed a significantly higher miscarriage rate [17]. Therefore, advanced paternal age may be associated with miscarriage, infertility, and birth defects [18].

Other non-hereditary factors implicated in embryonic chromosomal aberrations may include environmental factors. Prenatal exposure to environmental factors, such as drugs and pesticides, may increase the risk of birth defects or embryonic DNA damage [19, 20]. While several causes of teratogenesis have been identified, the etiopathogenetic mechanisms are not well characterized [20–22]. There is a paucity of epidemiological evidence pertaining to the risk factors for embryonic chromosomal aberrations in cases of spontaneous abortion. Miscarriage is a distressing event for the affected women and their families. Therefore, exploring the etiopathogenesis of spontaneous abortion may help inform interventions to protect the developing embryo and prevent miscarriage. The aim of this study was to assess the risk factors for spontaneous abortions with and without embryonic chromosomal aberrations by investigating the clinical and demographic characteristics of these cases.

## Materials and methods

### Study design and sample collection

This was a case-case study to estimate the risk factors for spontaneous abortions with and without embryonic chromosomal aberrations. Pregnant women diagnosed with spontaneous abortion were recruited at a tertiary general hospital in Kunming from October 2016 to March 2017. The hospital has a dedicated center for genetic diagnosis.

All cases of miscarriage were diagnosed based on ultrasound evidence of cessation of embryonal development or cessation of fetal cardiac activity during early pregnancy (gestational age, i.e., the time of embryo-fetal death estimated by ultrasound: < 14 weeks). The inclusion criteria were: singleton pregnancy with no obvious cause of abortion or teratogenicity such as uterine malformation, trauma, or abdominal x-ray examination [23].

**Fig. 1:**
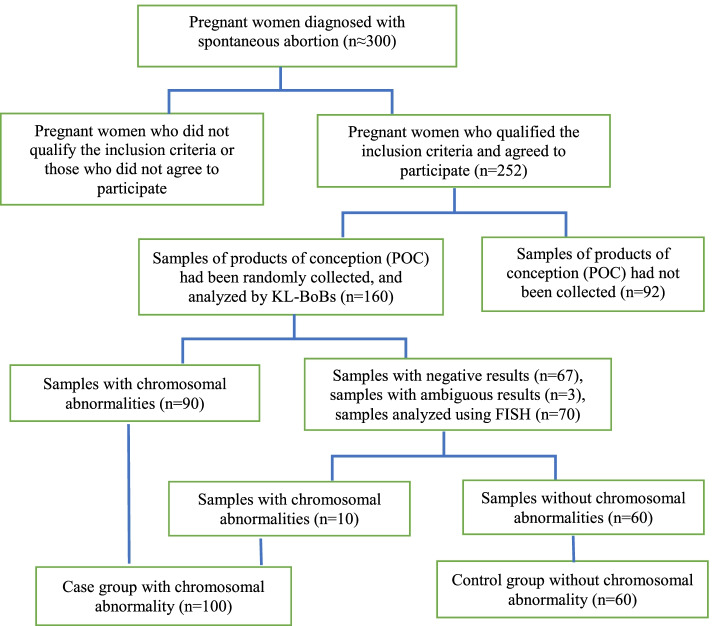
Flow diagram of patient enrollment and grouping

Initially, we approached about 300 pregnant women who were diagnosed with spontaneous abortions. Among them, 252 women agreed to participate in our study. A total of 160 samples of products of conception (POC) were randomly collected from the subject for chromosome analysis. The POC specimens were obtained by medical curettage procedure. The specimens were meticulously dissected from maternal deciduous tissue and rinsed with normal saline. First, KaryoLite BACs-on-Beads (KL-BoBs) assay was used to analyze the karyotype of the aborted tissues. Specimens with negative or ambiguous results were subjected to fluorescence in situ hybridization (FISH) method for detection. Pregnant women with normal fetal karyotype were assigned as the control group. A flow diagram of patient enrollment and grouping is shown in Fig. 1.

A self-designed questionnaire was used to collect data pertaining to potential causative factors for miscarriage, including maternal/paternal age, history of previous miscarriage, folic acid supplementation, and exposure to environmental factors (details of the questionnaire are added to the Supplementary file 1). In addition, blood samples were collected from pregnant women to assess the serum levels of homocysteine.

The study protocol was approved by the Ethics Committees of the Kunming Medical University. All women participated in the study voluntarily and provided written informed consent prior to their enrolment.

### KaryoLite™ BACs-on-beads™ assay

All 160 POC samples were processed using the KaryoLite™ BoBs™ assay kit (PerkinElmer, Turku, Finland) according to the manufacturer’s protocol. First, genomic DNA was extracted using the Trelief™ Animal Genomic DNA Kit (TSINGKE Biological Technology Inc., Beijing, China). Second, the extracted DNA was labeled with biotin, purified using a purification kit, and incubated overnight with the BoBs™ probes (fluorescently coded Luminex® beads with DNA probes). Thereafter, the hybridized beads were washed and bound to the reporter molecule (streptavidin phycoerythrin), and washed again. The DNA signals on the microbeads were measured using the Luminex® 200™ instrument system, and the experimental results were analyzed using the BoBsoft™ software. The detailed BoBs workflow is described elsewhere [24, 25].

### Fluorescence in situ hybridization (FISH) analysis

Due to its technical limitations, the BoBs assay is not appropriate for detection of polyploidy (e.g., triploid or tetraploid) or structural abnormalities [26]. Therefore, we used FISH technology to analyze the samples that yielded negative or ambiguous results with the KL-BoBs assay. FISH analysis was carried out using a commercial kit including five probes for chromosomes 21, 18, 13, X, and Y (GP Medical Technologies, Ltd., Beijing, China) according to the manufacturer’s instructions.

### Conventional karyotyping

To investigate the inherited embryonal chromosome aberrations, couples with chromosomal abnormalities in embryos (POC) were recalled for chromosome analysis. Approximately 3 mL venous blood samples were collected in an anticoagulant tube with heparin. Cell culture and G-banding analysis were conducted as per the conventional protocols [6, 7].

### Homocysteine (Hcy) measurement

Hcy level in maternal serum was detected by high performance liquid chromatography-tandem mass spectrometry (HPLC-MS/MS) (Waters, USA). Briefly, an aliquot of fasting serum was pretreated using a commercial kit (FOSUN PHARMA, Shanghai, China) according to the manufacturer’s instructions. Subsequently, 5 μL of the supernatant was injected into the HPLC-MA/MS system and separated by a C18 column (SHISEIDO, Japan). The calibration curve and quality control were achieved using the reagent in the kit.

### Statistical analysis

Data analyses were performed using SPSS 17.0 software. Association of fetal karyotype with continuous variables (parental age, homocysteine levels in maternal serum) was assessed using the independent-Samples *t* test and one-way analysis of variance (ANOVA). The Chi-Squared test was used to assess the association of fetal karyotype with categorical variables including folic acid supplementation (no, yes), previous miscarriages (0, ≥ 1), alcohol use (no, yes), and exposure to environmental factors (such as paint, noise, and passive smoking; detailed description of the related factors is provided in the Supplementary file 2). Finally, variables that showed a significant association on univariate analysis were included in multiple logistic regression models to identify factors that were different significantly between the two groups (Backward: LR, *P* < 0.10).

## Results

### Characteristics of the cases and karyotype analysis

The clinical and demographic characteristics of the study population are shown in Table 1 and Supplementary Table 1 (data not shown). The average age of the mothers was 30.8 years; most of the mothers had normal weight during early pregnancy, and more than a third had a previous history of spontaneous abortion.

**Table 1:** Characteristics of pregnant women with spontaneous abortion (*n* = 160)

	*n* (%) or Mean ± SD		*n* (%) or Mean ± SD
Maternal age (years)	30.8 ± 5.0	No. of previous miscarriages	
Paternal age (years)	32.6 ± 6.1	None	97 (60.6)
Ethnicity		One	49 (30.6)
Han	127 (79.4)	Two or more	14 (8.8)
Ethnic minorities	33 (20.6)	Folic acid supplementation	
Education		No	32 (20.0)
≤ Middle school	48 (30.0)	Yes	128 (80.0)
≥ College	112 (70.0)		
Place of residence		Serum homocysteine (μmol/L)	7.6 ± 2.5
Urban	127 (79.4)		
Suburban & rural	33 (20.6)	Maternal BMI (kg/m2)	21.9 ± 3.0

*SD* Standard deviation

**Fig. 2:**
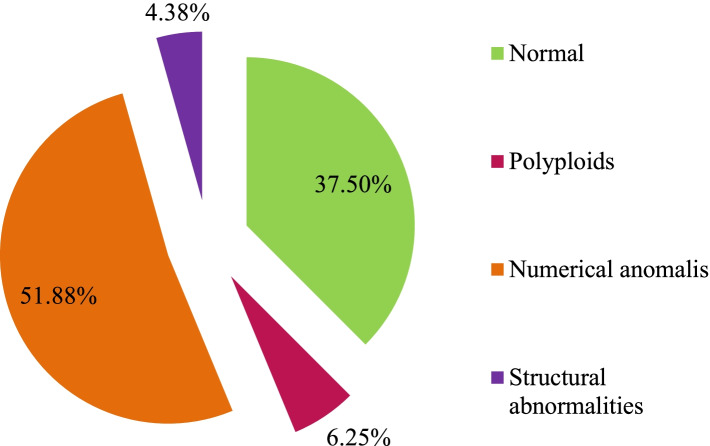
The proportion of chromosomal abnormalities in POC specimens (*n* = 160)

**Fig. 3:**
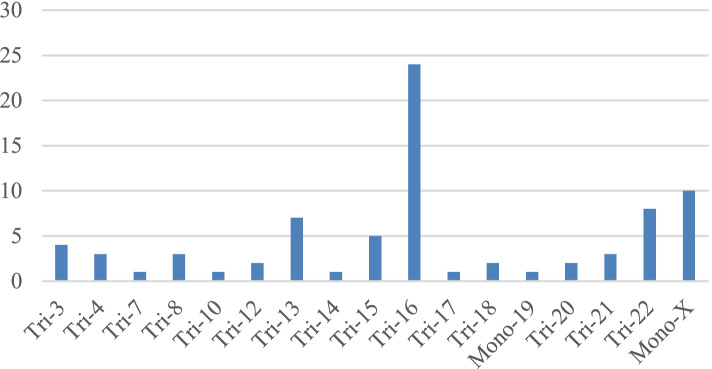
Distribution of aneuploidies involving a single chromosome

The KL-BoBs assay was performed in all spontaneous abortions, and chromosomal abnormalities were detected in 90 of 160 cases. The remaining 67 negative cases and 3 ambiguous cases were analyzed by FISH; among these, 7 cases were triploid, and 3 cases were tetraploid. The overall rate of chromosomal abnormalities in POC specimens was 62.5% (100/160). Among these, 6.3% (10/160) were polyploid, 51.9% (83/160) were abnormalities in chromosome number, and 4.4% (7/160) were chromosomal structural abnormalities, including 1 case with both chromosome number and structural abnormalities (Fig. 2, Supplementary Table 2). Among the cases with abnormal number of chromosomes, 78 cases involved a single chromosome, and 5 cases involved two chromosomes; of the former, the most common aneuploidies were trisomy 16 (*n* = 24), followed by monosomy X (*n* = 10), trisomy 22 (*n* = 8), and trisomy 13 (*n* = 7) (Fig. 3).

### Risk factors for chromosome aberration in aborted embryos

#### Genetic factors

A total of 90 couples with aneuploid or structurally abnormal chromosomes in embryos were recalled for cytogenetic analysis. Among them, 15 couples were lost to follow up (including 9 cases of aneuploidies involving group D or G chromosomes and 6 cases of aneuploidies involving other chromosomes). One case of structural abnormality was found to be derived from maternal balanced translocation (Supplementary Table 2).

#### Clinical background and environmental factors

**Fig. 4:**
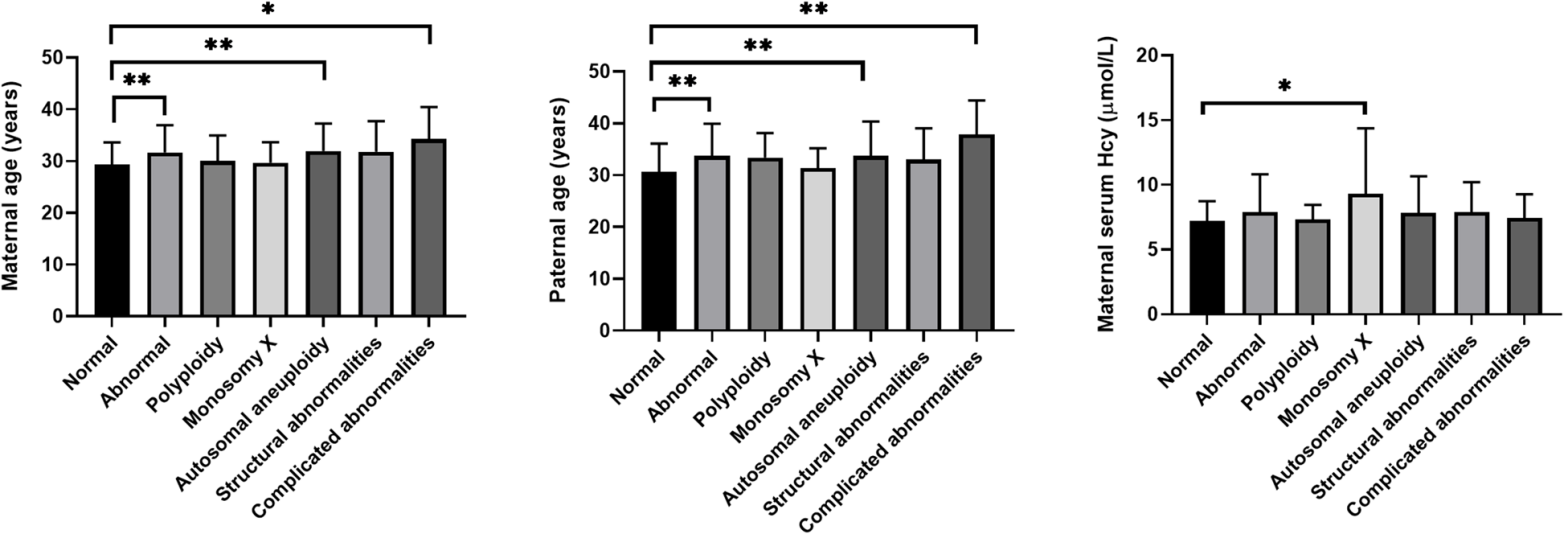
Association of fetal karyotype with parental clinical background factors (continuous variables)

The association of karyotype in the aborted embryos and the parental clinical background or exposure to environmental factors are shown in Fig. 4 (for continuous variables) and Table 2 (for categorical variables). Women with abnormal embryonic chromosomes were significantly older than women with normal embryonic chromosomes (*P* < 0.01), especially those with autosomal aneuploidy and complex aberrations (abnormalities involving ≥2 chromosomes). In addition, the paternal age of the group with abnormal fetal chromosomes was greater than that of the group with normal fetal chromosomes (*P* < 0.01). Due to the small number of cases, we did not find a significant difference in the parental age of embryos with normal karyotype and embryos with polyploid or X monosomy (Fig. 4).

**Table 2:** Association of fetal chromosomal abnormalities with maternal characteristics (categorical variables)

	Normal	Abnormal	*P*	Crude OR (95% CI)
Folic acid supplementation				
No	13 (21.7)	19 (19.0)		
Yes	47 (78.3)	81 (81.0)	0.683	1,18 (0.53, 2.60)
Previous miscarriages (n)				
< 1	**30 (50.0)**	**66 (66.0)**		
≥ 1	**30 (50.0)**	**34 (34.0)**	**0.046**	0.52 (0.27, 0.99)*
Exposure to paint				
No	59 (98.3)	92 (92.0)		
Yes	1 (1.7)	8 (8.0)	0.184	5.13 (0.63, 42.08)
Perceived noises				
No	**46 (76.7)**	**92 (92.0)**		
Yes	**14 (23.3)**	**8 (8.0)**	**0.006**	0.29 (0.11, 0.73)**
Pesticide exposure				
No	53 (88.3)	91 (91.0)		
Yes	7 (11.7)	9 (9.0)	0.586	0.75 (0.26, 2.13)
Repellent exposure				
No	50 (83.3)	84 (84.0)		
Yes	10 (16.7)	16 (16.0)	0.912	0.95 (0.40, 2.26)
Passive smoking				
No	26 (43.3)	45 (45.0)		
Yes	34 (56.7)	55 (55.0)	0.837	0.94 (0.49, 1.78)
Alcohol use				
No	57 (95.0)	96 (96.0)		
Yes	3 (5.0)	4 (4.0)	1.000	0.79 (0.17, 3.67)
Exposure to PAH				
No	48 (80.0)	89 (89.0)		
Yes	12 (20.0)	11 (11.0)	0.116	0.49 (0.20, 1.20)

Bold font indicates statistical significance; the 95% CI does not include 1

*Abbreviations*: *PAH* Polycyclic aromatic hydrocarbons

^*^indicates *P* < 0.05

^**^indicates *P* < 0.01

Serum Hcy level in women with abnormal embryonic chromosomes was greater than that of women with normal embryonic chromosomes, although the difference was not statistically significant (*P* = 0.081). Regarding the individual group comparisons, only monosomy X was significantly related to the Hcy level in maternal serum (*P* = 0.014). Folic acid supplementation showed no significant association with fetal chromosomal abnormalities (*P* = 0.683).

Women with ≥1 previous miscarriage had a significantly lower odds ratio for abnormal karyotypes than women without previous miscarriage [crude odds ratio (OR) = 0.52; 95% confidence interval (CI), 0.27–0.99, *P* = 0.046]. The odds ratio of fetal chromosomal aberration in women with self-perceived exposure to noise was lower than that in women who were not exposed (crude OR = 0.29; 95% CI, 0.11–0.73, *P* = 0.006). Fetal chromosomal aberrations showed no significant association with exposure to other environmental factors (such as paint, pesticide, PAH, alcohol, or passive smoking) (Table 2).

On multivariate logistic analyses, the risk of abortus with chromosomal aberration was positively associated with paternal age (OR = 1.11; 95% CI, 1.03–1.18, *P* = 0.003) and maternal serum Hcy level (OR = 1.28; 95% CI, 1.02–1.60, *P* = 0.037). The odds ratio for fetal chromosomal aberrations was significantly decreased in women exposed to noise (OR = 0.27; 95% CI, 0.10 to 0.76, *P* = 0.013), but increased in women exposed to paint (OR = 6.89; 95% CI, 0.66 to 71.81, *P* = 0.106) (Table 3).

**Table 3:** Results of multivariate logistic analyses showing the association of parental characteristics with fetal chromosomal aberrations

	Chromosomal abnormality OR (95% CI)	*P*
Paternal age (years, continuous)	**1.11 (1.03, 1.18)**	0.003**
Serum homocysteine (μmol/L, continuous)	**1.28 (1.02, 1.60)**	0.037*
Previous miscarriages (n)		
≥ 1 vs 0	0.53 (0.26, 1.09)	0.083
Exposure to paint		
Yes vs No	6.89 (0.66, 71.81)	0.106
Perceived noises		
Yes vs No	**0.27 (0.10, 0.76)**	0.013*

All variables were introduced into the model and selected with a Backward: LR strategy, remove at *P* > 0.10

Bold font indicate statistical significance; the 95% CI does not include 1

^*^indicates *P* < 0.05

^**^indicates *P* < 0.01

## Discussion

Consistent with previous research [3], most of the aborted embryos in this study had chromosomal abnormalities. Parental age may be an independent risk factor for fetal chromosomal abnormalities, especially for autosomal aneuploidies and complex aberrations. After adjusting for some potential confounding factors, the risk of embryonic chromosomal aberrations showed a positive correlation with paternal age, but not with maternal age. The reason may be that as the mother aged, the risk of fetal chromosomal aberrations increased, and the risk of unexplained spontaneous abortion increased as well [27].

Similar to the earlier reports [3, 28], the risk of polyploidy was not positively correlated with maternal age. Polyploidy, known as the endoreplication of genomic DNA [29], has been found to increase the adaptive potential of organisms exposed to stressful conditions [30]. However, polyploidization in embryonic DNA has serious consequences, and few can survive past the first trimester [31].

The incidence of monosomy X showed no association with maternal age, which is consistent with previous reports [3, 28]. Similarly, the incidence of monosomy X did not differ by paternal age, although in a previous study, monosomy X was found more likely to be caused by paternal chromosome loss [32]. Nevertheless, we found that the serum level of Hcy in mothers with monomer X was higher than that in mothers with normal karyotype. In addition, the odds ratio for embryonic chromosomal abnormalities increased with the maternal serum Hcy level. Consistent with previous reports, high concentrations of Hcy may induce DNA damage and increase the risk of genomic instability in humans [33]. Hcy is an intermediate product of folate metabolism; however, we found no correlation between folate supplementation and embryonic chromosomal aberrations. This may be attributable to inter-individual variability with respect to folate uptake and metabolism [13]. In addition, we did not assess the timing of initiation of folic acid supplementation and the amount of supplementation, which may affect the effectiveness of folic acid supplementation [12, 34].

There is no clear consensus on the association between obstetric history and embryonic karyotype. In a study by Ozawa et al., compared with women with < 2 previous miscarriages, women with ≥2 previous miscarriages had a lower incidence of chromosomal abnormalities in the aborted embryos [3]. For parents with normal karyotypes, the incidence of abnormal embryo karyotypes decreased significantly with the number of previous miscarriages [9, 35], implying an unascertained maternal cause for spontaneous abortion [27]. However, other researchers have found no correlation between the number of previous miscarriages and chromosomal aberration in the aborted embryo [36]. However, the study did not clarify whether chromosomal aberrations in embryos were inherited from balanced rearrangements in parental chromosome.

Parental chromosomal abnormality is an important genetic cause of spontaneous miscarriage. In this study, only one case of structural abnormality was found to be derived from maternal balanced chromosomal translocation. In accordance with previous studies, most miscarriages that occurred during early pregnancy were due to non-inherited chromosomal aneuploidy [1, 2], suggesting the potential involvement of environmental factors in embryonic chromosomal teratogenesis and/or spontaneous abortion.

In this case-case study, the incidence of embryonic chromosomal aberrations showed a negative correlation with prenatal exposure to noise. The result indicated that prenatal exposure to noise may be related to spontaneous abortion in embryos with normal karyotype. Animal experiments have shown that noise may have a direct effect on developing animals by increasing the embryo absorption and decreasing live births per litter [37, 38]. In addition, Noise may have an indirect impact by reducing the uteroplacental blood flow and increasing the release of catecholamines (one of the stress hormones), which may induce fetal hypoxia and abnormal embryogenesis [39]. Till date, a few epidemiologic studies have investigated the association between noise exposure and spontaneous abortion. However, the quality of evidence of this association is very low [40, 41].

Women with a history of exposure to paint showed higher incidence of fetal chromosomal aberrations, although, the association was not statistically significant; this may be attributable to the small sample size. Thus, exposure to paint may increase the risk of embryonic chromosomal aberrations. Volatile organic compounds (VOC) emitted from paint may cause chromosomal aberrations [42, 43].

This was a small-scale study to estimate the risk factors of spontaneous abortions with and without embryonic chromosomal aberrations. In this study, we used both KL-BoBs and FISH technology to analyze the aborted POC tissues, which avoided the influence of cell culture failure and maternal cell contamination on the karyotype results [3, 26]. This approach helped overcome the limitations of BoBs technique in analyzing polyploidy [26, 44], and improved the detection rate and accuracy of the results. There are three main limitations of this study. First, this combined method is unable to detect chromosomal microdeletions or microduplications in regions not covered by the kit and balanced rearrangements [24, 44]. Second, we did not perform next generation sequencing (NGS) and chromosomal microarray analysis (CMA) for copy number variations (CNVs) and/or single nucleotide variants (SNVs) in euploid miscarriages [45–47]. This may have led to misclassification in the division of the case group and the control group. However, possible pathogenic CNVs were detected in ∼2% of miscarriages, and a large number of miscarriages had CNVs of unknown significance [45, 47]. Mutations in genes involved in embryo implantation, angiogenesis, coagulation, immunological function response, and fetal growth, may contribute to or predispose to pregnancy loss [45, 46, 48]. The clinically useful SNVs need to be validated by in vitro/in vivo functional tests. Third, some potential factors related to spontaneous abortion or embryonic chromosomal aberrations were not included in this study, such as assisted reproduction, thrombophilic disorders, immune dysfunction, and exposure to other environmental factors.

## Conclusion

The main cause of spontaneous abortion in our cohort was non-hereditary embryo chromosomal abnormalities. Paternal age, reproductive history, maternal Hcy levels, and exposure to environmental factors may be associated with spontaneous abortion with or without fetal chromosomal aberrations. Our findings may provide scientific evidence for prenatal care and genetic consultation.

## Supplementary Information

**Additional file 1.** Details of the questionnaire.

**Additional file 2.** Detailed description of the related factors.

**Additional file 3: Supplemental Table 1.** Association of fetal chromosomal abnormalities with parental characteristics (data not shown, *n* = 160).

**Additional file 4: Supplemental Table 2.** Details of structural and complicated abnormalities detected in miscarriages.

## Data Availability

The datasets generated and/or analysed during the current study are not publicly available due [patient/participant anonymity] but are available from the corresponding author on reasonable request.

## Abbreviations

KL-BoBs: KaryoLite BACs-on-Beads
FISH: Fluorescence in situ hybridization
Hcy: Homocysteine
HPLC-MS/MS: High performance liquid chromatography-tandem mass spectrometry
POC: Products of conception
BMI: Body mass index
SD: Standard deviation
OR: Odds ratio
95% CI: 95% confidence interval
PAH: Polycyclic aromatic hydrocarbons
VOC: Volatile organic compounds
NGS: Next generation sequencing
CMA: Chromosomal microarray analysis
CNVs: Copy number variations
SNVs: Single nucleotide variants

## References

[1] Larsen EC, Christiansen OB, Kolte AM, Macklon N (2013). New insights into mechanisms behind miscarriage. BMC Med.

[2] Branch DW, Gibson M, Silver RM (2010). Clinical practice. Recurrent miscarriage. N Engl J Med.

[3] Ozawa N, Ogawa K, Sasaki A, Mitsui M, Wada S, Sago H (2019). Maternal age, history of miscarriage, and embryonic/fetal size are associated with cytogenetic results of spontaneous early miscarriages. J Assist Reprod Genet.

[4] Wang BT, Chong TP, Boyar FZ, Kopita KA, Ross LP, El-Naggar MM (2014). Abnormalities in spontaneous abortions detected by G-banding and chromosomal microarray analysis (CMA) at a national reference laboratory. Mol Cytogenet.

[5] Tunç E, Tanrıverdi N, Demirhan O, Süleymanova D, Çetinel N (2016). Chromosomal analyses of 1510 couples who have experienced recurrent spontaneous abortions. Reprod BioMed Online.

[6] Gada Saxena S, Desai K, Shewale L, Ranjan P, Saranath D (2012). Chromosomal aberrations in 2000 couples of Indian ethnicity with reproductive failure. Reprod BioMed Online.

[7] Fan HT, Zhang M, Zhan P, Yang X, Tian WJ, Li RW. Structural chromosomal abnormalities in couples in cases of recurrent spontaneous abortions in Jilin Province, China. Genet Mol Res. 2016;15(1). 10.4238/gmr.15017443. 10.4238/gmr.15017443 26909916

[8] Van Dyke DL, Weiss L, Roberson JR, Babu VR (1983). The frequency and mutation rate of balanced autosomal rearrangements in man estimated from prenatal genetic studies for advanced maternal age. Am J Hum Genet.

[9] Suzumori N, Sugiura-Ogasawara M (2010). Genetic factors as a cause of miscarriage. Curr Med Chem.

[10] Gaiday AN, Tussupkaliyev AB, Bermagambetova SK, Zhumagulova SS, Sarsembayeva LK, Dossimbetova MB (2018). Effect of homocysteine on pregnancy: a systematic review. Chem Biol Interact.

[11] Serapinas D, Boreikaite E, Bartkeviciute A, Bandzeviciene R, Silkunas M, Bartkeviciene D (2017). The importance of folate, vitamins B6 and B12 for the lowering of homocysteine concentrations for patients with recurrent pregnancy loss and MTHFR mutations. Reprod Toxicol.

[12] Mao YY, Yang L, Li M, Liu J, Zhu QX, He Y, et al. Periconceptional folic acid supplementation and the risk of spontaneous abortion among women who prepared to conceive: impact of supplementation initiation timing. Nutrients. 2020;12(8). 10.3390/nu12082264. 10.3390/nu12082264 PMC7469034 32751085

[13] DeVos L, Chanson A, Liu Z, Ciappio ED, Parnell LD, Mason JB (2008). Associations between single nucleotide polymorphisms in folate uptake and metabolizing genes with blood folate, homocysteine, and DNA uracil concentrations. Am J Clin Nutr.

[14] Enciso M, Sarasa J, Xanthopoulou L, Bristow S, Bowles M, Fragouli E (2016). Polymorphisms in the MTHFR gene influence embryo viability and the incidence of aneuploidy. Hum Genet.

[15] Sharma R, Agarwal A, Rohra VK, Assidi M, Abu-Elmagd M, Turki RF (2015). Effects of increased paternal age on sperm quality, reproductive outcome and associated epigenetic risks to offspring. Reprod Biol Endocrinol.

[16] Das M, Al-Hathal N, San-Gabriel M, Phillips S, Kadoch IJ, Bissonnette F (2013). High prevalence of isolated sperm DNA damage in infertile men with advanced paternal age. J Assist Reprod Genet.

[17] Robinson L, Gallos ID, Conner SJ, Rajkhowa M, Miller D, Lewis S (2012). The effect of sperm DNA fragmentation on miscarriage rates: a systematic review and meta-analysis. Hum Reprod.

[18] Brandt JS, Cruz Ithier MA, Rosen T, Ashkinadze E (2019). Advanced paternal age, infertility, and reproductive risks: a review of the literature. Prenat Diagn.

[19] Scheuerle AE, Aylsworth AS (2016). Birth defects and neonatal morbidity caused by teratogen exposure after the embryonic period. Birth Defects Res A Clin Mol Teratol.

[20] Alvarado-Hernandez DL, Montero-Montoya R, Serrano-García L, Arellano-Aguilar O, Jasso-Pineda Y, Yáñez-Estrada L (2013). Assessment of exposure to organochlorine pesticides and levels of DNA damage in mother-infant pairs of an agrarian community. Environ Mol Mutagen.

[21] Toufaily MH, Westgate MN, Lin AE, Holmes LB (2018). Causes of congenital malformations. Birth Defects Res.

[22] Baldacci S, Gorini F, Santoro M, Pierini A, Minichilli F, Bianchi F (2018). Environmental and individual exposure and the risk of congenital anomalies: a review of recent epidemiological evidence. Epidemiol Prev.

[23] Xu Q, Zhu B, Dong X, Li S, Song X, Xiao X (2020). Pyrethroid pesticide exposure during early pregnancy and birth outcomes in Southwest China: a birth cohort study. J Toxicol Sci.

[24] Grati FR, Vialard F, Gross S (2015). BACs-on-beads™ (BoBs™) assay for the genetic evaluation of prenatal samples and products of conception. Methods Mol Biol.

[25] Campos-Galindo I, García-Herrero S, Martínez-Conejero JA, Ferro J, Simón C, Rubio C (2015). Molecular analysis of products of conception obtained by hysteroembryoscopy from infertile couples. J Assist Reprod Genet.

[26] Pérez-Durán J, Nájera Z, Trujillo-Cabrera Y, Martín-Saro M, García-Latorre E, Escarcega-Preciado J (2015). Aneusomy detection with Karyolite-Bac on beads® is a cost-efficient and high throughput strategy in the molecular analyses of the early pregnancy conception losses. Mol Cytogenet.

[27] Magnus MC, Wilcox AJ, Morken NH, Weinberg CR, Håberg SE (2019). Role of maternal age and pregnancy history in risk of miscarriage: prospective register based study. BMJ..

[28] Eiben B, Bartels I, Bähr-Porsch S, Borgmann S, Gatz G, Gellert G (1990). Cytogenetic analysis of 750 spontaneous abortions with the direct-preparation method of chorionic villi and its implications for studying genetic causes of pregnancy wastage. Am J Hum Genet.

[29] Fox DT, Duronio RJ (2013). Endoreplication and polyploidy: insights into development and disease. Development..

[30] Van de Peer Y, Mizrachi E, Marchal K (2017). The evolutionary significance of polyploidy. Nat Rev Genet.

[31] Toufaily MH, Roberts DJ, Westgate MN, Holmes LB (2016). Triploidy: variation of phenotype. Am J Clin Pathol.

[32] Hassold T, Benham F, Leppert M (1988). Cytogenetic and molecular analysis of sex-chromosome monosomy. Am J Hum Genet.

[33] Picerno I, Chirico C, Condello S, Visalli G, Ferlazzo N, Gorgone G (2007). Homocysteine induces DNA damage and alterations in proliferative capacity of T-lymphocytes: a model for immunosenescence?. Biogerontology..

[34] Roffman JL (2018). Neuroprotective effects of prenatal folic acid supplementation: why timing matters. JAMA Psychiatry.

[35] Ogasawara M, Aoki K, Okada S, Suzumori K (2000). Embryonic karyotype of abortuses in relation to the number of previous miscarriages. Fertil Steril.

[36] Goldstein M, Svirsky R, Reches A, Yaron Y (2017). Does the number of previous miscarriages influence the incidence of chromosomal aberrations in spontaneous pregnancy loss?. J Matern Fetal Neonatal Med.

[37] Meyer RE, Aldrich TE, Easterly CE (1989). Effects of noise and electromagnetic fields on reproductive outcomes. Environ Health Perspect.

[38] Rasmussen S, Glickman G, Norinsky R, Quimby FW, Tolwani RJ (2009). Construction noise decreases reproductive efficiency in mice. J Am Assoc Lab Anim Sci.

[39] Ristovska G, Laszlo HE, Hansell AL (2014). Reproductive outcomes associated with noise exposure - a systematic review of the literature. Int J Environ Res Public Health.

[40] McDonald AD, Armstrong B, Cherry NM, Delorme C, Diodati-Nolin A, McDonald JC (1986). Spontaneous abortion and occupation. J Occup Med.

[41] Dzhambov AM, Dimitrova DD, Dimitrakova ED (2014). Noise exposure during pregnancy, birth outcomes and fetal development: meta-analyses using quality effects model. Folia Med (Plovdiv).

[42] Wei W, Wang SX, Hao JM (2009). Estimation and forecast of volatile organic compounds emitted from paint uses in China. Huan Jing Ke Xue.

[43] Xie J, Liu YH, Li LF, Wu YM (2010). A clinical analysis of fetal chromosomal aberration induced by paint and hair dye. Nan Fang Yi Ke Da Xue Xue Bao.

[44] Li C, Chen B, Zheng J, Cheng L, Song T, Guo F (2019). Prenatal diagnosis of BACs-on-beads assay in 3647 cases of amniotic fluid cells. Reprod Sci.

[45] Qiao Y, Wen J, Tang F, Martell S, Shomer N, Leung PC (2016). Whole exome sequencing in recurrent early pregnancy loss. Mol Hum Reprod.

[46] Colley E, Hamilton S, Smith P, Morgan NV, Coomarasamy A, Allen S (2019). Potential genetic causes of miscarriage in euploid pregnancies: a systematic review. Hum Reprod Update.

[47] Pauta M, Grande M, Rodriguez-Revenga L, Kolomietz E, Borrell A (2018). Added value of chromosomal microarray analysis over karyotyping in early pregnancy loss: systematic review and meta-analysis. Ultrasound Obstet Gynecol.

[48] Quintero-Ronderos P, Laissue P (2020). Genetic variants contributing to early recurrent pregnancy loss etiology identified by sequencing approaches. Reprod Sci.

