# Supplementary material for: Factors associated with fetal karyotype in spontaneous abortion: a case-case study
Source: BMC Pregnancy Childbirth. 2022 Apr 14;22:320. doi: 10.1186/s12884-022-04491-8 (PMC9012016; doi:10.1186/s12884-022-04491-8)
Supplement: Supplementary file 1 — Additional file 1. Details of the questionnaire. [file 12884_2022_4491_MOESM1_ESM.doc]

No. □·□·□□□

**Environmental Health Survey for Pregnant Women**

| Investigator: | Record date: |
| --- | --- |

**A. Information on maternal health and family history**

**A1.** Maternal height: cm; Maternal weight: kg.

**A2.** Start date of last menstrual period: .

**A3.** How many times are you pregnant? times.

**A4.** How many children did you deliver?

① none ② one and more

**A5**. Have you had any of the following conditions in the past? (You can choose more than one, and indicate the number of times, please fill in item by item, if no, please fill in "0")

① Spontaneous abortion times ② Induced abortion times

③ Fetal death/stillbirth times ④ Molar pregnancy times

⑤ Induced labor due to fetal abnormalities in the second or third trimesters, times，

⑥ No ⑦ I don't know

**A6.** Do you suffer from the following diseases? (Multiple choice)

① Hypertension ② Diabetes ③ Thyroid disease ④ Congenital heart disease

⑤ Tumor (indicate the specific name) ⑥ Uterine malformation (indicate the type)

⑦ Uterine fibroids (specify location and size)

⑧ Other diseases: ⑨ None ⑩ Don’t know

**A7.** Are there any of the following genetic diseases in your and/or your husband family (three direct generations)? (Multiple choice)

① Congenital heart disease ② Congenital mental retardation

③ Other genetic diseases: ④ None ⑤ Don't know

**A8.** Have you been supplemented with folic acid or nutrients containing folic acid since three months before this pregnancy?

① No, I never had a supplement ② Yes, I had a supplement

**A9.** Have you taken any medication in the past six months?

① No ② Yes, I have taken (the medicine name)

**B. Pesticide exposure**

**B1.** Have you been exposed to household pesticides in your home/office in the past six months?

① No ② Yes，what kind of household pesticides have you been exposed to:

**B2.** Have you been exposed to repellents in your home/office in the past six months?

① No ② Yes，what kind of repellents have you been exposed to:

**B3.** Have you been exposed to agricultural pesticides in the past six months?

① No ② Yes，what kind of agricultural pesticides have you been exposed to:

**B4.** One month before your conception, has your husband been exposed to household insecticides?

① No ② Yes，what kind of household pesticides has he been exposed to:

**B5.** One month before your conception, has your husband been exposed to repellents?

① No ② Yes，what kind of repellents has he been exposed to:

**B6.** One month before your conception, has your husband been exposed to agricultural insecticides?

① No ② Yes，what kind of agricultural pesticides has he been exposed to:

**C. Living/working conditions and lifestyle**

**C1.** Where do you live? ① Urban areas ② Suburbs ③ Rural areas

**C2.** Are there any of the following conditions near your home? (Multiple choice)

① Barbecue stall, distance meters ② Road, distance meters

③ Fruit/vegetable/flower planting areas, distance meters

④ Thermal power plants/smelters/cement plants, distance meters

⑤ Others, distance meters.

**C3.** Do you cook before and/or after pregnancy?

① Never do it ② Less than 3 days a week ③ More than 3 days a week ④ Do it every day

**C4.** What exhaust equipment is installed in your kitchen?

① No ② Exhaust fan ③ Exhaust hood ④ Chimney

**C5.** The main cooking methods in your family are:

① frying/barbecuing ② stir-frying ③ steaming/boiling

**C6.** Do you use plastic containers for hot food or water?

① No ②Yes, times a week.

**C7.** Do you use plastic containers to heat food or water?

① No ② Yes, times a week.

**C8.** Which fruits do you usually eat? (Multiple choice)

① Apple ② Banana ③ Pear ④ Orange/orange ⑤ Watermelon ⑥ Cantaloupe

⑦ Small tomato ⑧ Others .

**C9.** Which vegetables do you usually eat? (Multiple choice)

① Water spinach ② Cucumber ③ Cauliflower ④ Chinese cabbage ⑤ Tomato

⑥ Potato ⑦ Leek ⑧ Other .

**C10.** What method do you use to clean fruits and vegetables? (Multiple choice)

① Do not wash, eat directly ② Eat after washing with water

③ Soak and wash with water/salt water ④ Soak and wash with rice water

⑤ Soak and wash with flour water ⑥ Soak and wash with soda water

⑦ Soak and wash with other methods or other detergents .

**C11.** Have you been exposed to any of the following substances since 3 months before this pregnancy? (Multiple choice)

① CT or X-ray examination (except on the abdomen)

② Alcohol use, drinking ml, alcohol content % ③ Exposure to paint

④ Exposure to other environmental factors .

**C12.** Has your husband been exposed to the following substances one month before your conception? (Multiple choice)

1. CT or X-ray examination (except on the abdomen)

② Alcohol use, drinking ml, alcohol content %

③ Exposure to paint ④ Exposure to other environmental factors .

**C13.** Do you think there is noise in your work and/or living environment?

① No; ②Yes, the noise comes from .

**C14.** Have you smoked from 3 months before pregnancy to now?

① Never smoked ② Smoking before pregnancy, quit smoking after pregnancy

③ I have smoked

**C15.** Did your husband smoke?

① Never smoked ② Used to smoke, and now have quit smoking for years

③ Smoking for years

**C16.** Since 3 months before your pregnancy, has anyone smoked by your side?

① No ② Yes

**C17.** Have you had a job in the past six months? ① No ②Yes

**C18.** What kind of work have you engaged in in the past six months? (Select one by one)

① Chef/catering ② Oil locomotive driver/traffic police

③ Pesticide production/pesticide sales ④ Agricultural production

⑤ Indoor and outdoor decoration ⑥ Medical radiology/nuclear medicine ⑦ Others

**C19.** Have your husband had a job in the past six months? ① No ②Yes

**C20.** What kind of work have your husband engaged in in the past six months? (Select one by one)

① Chef/catering ② Oil locomotive driver/traffic police

③ Pesticide production/pesticide sales ④ Agricultural production

⑤ Indoor and outdoor decoration ⑥ Medical radiology/nuclear medicine ⑦ Others

**D. Demographic sociological characteristics**

**D1.** Your age is years， and your ethnicity is .

**D2.** Your highest education:

① Junior high school and below ② High school/vocational school/technical school

③ College and undergraduate ④ Postgraduate ⑤ Refused to answer

**D3.** Your husband’s age is year, and your husband’s ethnicity is .

**D4.** Your husband’s highest education:

① Junior high school and below ② High school/vocational school/technical school

③ College and undergraduate ④ Postgraduate ⑤ Refused to answer

**D5.** How much is your monthly household income?

① <2,000 yuan ② 2,000–5000 yuan ③ 5000–10,000 yuan ④ 10,000–15000 yuan

⑤ 15,000–20,000 yuan ⑥ 20,000–30,000 yuan ⑦ 30,000 yuan or more

⑧ Refuse to answer
