# Supplementary material for: Factors associated with fetal karyotype in spontaneous abortion: a case-case study
Source: BMC Pregnancy Childbirth. 2022 Apr 14;22:320. doi: 10.1186/s12884-022-04491-8 (PMC9012016; doi:10.1186/s12884-022-04491-8)
Supplement: Supplementary file 2 — Additional file 2. Detailed description of the related factors. [file 12884_2022_4491_MOESM2_ESM.docx]

**Detailed description of the related factors**

**Exposure to paint,** means exposure to paint in the work and /or living environments, mainly including decorative paint and wood paint.

**Perceived noises** are subjectively reported by pregnant women, which means exposure to all noises in work and /or living environments.

**Pesticide exposure** means that someone had used pesticides (including household pesticides and agricultural pesticides) in the environment where pregnant women worked and/or lived, mainly other people had used them.

**Repellent exposure** means that someone (including pregnant women themselves and others) had used repellent (mainly to repel mosquitoes) in the environment where pregnant women worked and/or lived.

**Passive smoking** means that colleagues, family members or friends of the pregnant women smoked by their side.

**Alcohol use** means that the pregnant woman consumed alcohol or alcoholic beverages, including beer, wine, liquor, or other alcoholic beverages. During the recruitment of pregnant women, only a few women reported drinking small amounts of alcohol.

**Exposure to polycyclic aromatic hydrocarbons (PAH)** refers to working in a restaurant, working as a driver/traffic policeman, or cooking every day in a kitchen without ventilation.

**Folic acid supplementation** includes supplementing folic acid and/or supplementing nutrients containing folic acid.

**The exposure time** for pregnant women is within the 6 months immediately preceding the recruitment, or from 3 months before conception to the time of recruitment. The pregnant women in the case group and the control group were interviewed using the same questionnaire.

**Thyroid diseases** include hypothyroidism, hyperthyroidism, and thyroid tumors. And all pregnant women who were diagnosed with thyroid disease received standardized treatment.
